# Correction: Site- and stereoselective silver-catalyzed intramolecular amination of electron-deficient heterobenzylic C–H bonds

**DOI:** 10.1039/d5sc90120k

**Published:** 2025-05-30

**Authors:** Tuan Anh Trinh, Stanislav Cherempei, Daniel S. Rampon, Jennifer M. Schomaker

**Affiliations:** a Department of Chemistry, University of Wisconsin–Madison Madison Wisconsin 53706 USA schomakerj@chem.wisc.edu

## Abstract

Correction for ‘Site- and stereoselective silver-catalyzed intramolecular amination of electron-deficient heterobenzylic C–H bonds’ by Tuan Anh Trinh *et al.*, *Chem. Sci.*, 2025, **16**, 4796–4805, https://doi.org/10.1039/D4SC08757G.

The authors regret that in the original paper, the incorrect funding number was given in the Acknowledgements section to acknowledge the funding received by Jennifer M. Schomaker from the National Science Foundation. The Acknowledgements should read: J. M. S. is grateful to NSF (CHE-2247217).

The Royal Society of Chemistry apologises for these errors and any consequent inconvenience to authors and readers.

